# Heart Rate Variability in Newborns From Diabetic Mothers: Clinical Application and Significance

**DOI:** 10.1111/anec.70173

**Published:** 2026-03-28

**Authors:** Virginia Beretta, Laura Cannavò, Sebastiano Ravenda, Chiara Petrolini, Vincenzo Raitano, Sabrina Moretti, Valentina Dell'Orto, Serafina Perrone

**Affiliations:** ^1^ Neonatology Unit, Department of Medicine and Surgery, University of Parma Pietro Barilla Children's Hospital Parma Italy; ^2^ Pediatric Intensive Care Unit University Hospital of Verona Verona Italy; ^3^ Stress Physiology Laboratory, Department of Chemistry Life Sciences and Environmental Sustainability – University of Parma Parma Italy

**Keywords:** autonomic nervous system, fetal programming, gestational diabetes mellitus, heart rate variability, newborn

## Abstract

Gestational diabetes mellitus (GDM) is one of the most common pregnancy complications, arising from inadequate insulin secretion and increased maternal insulin resistance. Globally, GDM affects up to 16%–17% of pregnancies, with higher prevalence in low‐ and middle‐income countries. Poorly controlled maternal hyperglycemia increases the risk of congenital malformations, intrauterine death, macrosomia, and metabolic disturbances, and contributes to long‐term vulnerabilities including obesity, type 2 diabetes, and cardiovascular disease in offspring. Beyond its metabolic consequences, GDM is increasingly recognized as a prenatal condition capable of shaping fetal developmental trajectories. Emerging evidence indicates that maternal hyperglycemia influences the maturation of the autonomic nervous system (ANS), with measurable effects on neonatal heart rate variability (HRV), a widely used non‐invasive proxy of autonomic function, although not a direct measure of sympathetic and parasympathetic activity. Findings from fetal magnetocardiography and neonatal electrocardiography show that exposure to GDM is associated with altered HRV profiles, such as sympathetic predominance, reduced autonomic flexibility, or atypical vagal modulation, depending on perinatal physiology. These patterns suggest that ANS dysregulation can originate in utero and persist into the early postnatal period. This educational review examines the biological and pathophysiological mechanisms linking maternal hyperglycemia to impaired ANS development in fetuses and newborns. Alongside epidemiological and clinical data on infants born to mothers with GDM, it synthesizes recent evidence positioning HRV as a potential early biomarker of autonomic dysregulation. Ultimately, GDM's influence extends beyond glycemic imbalance, affecting the neurobiological foundations of autonomic function and potentially identifying neonates at risk for later cardiometabolic complications.

Risk factors for GDM contribute to a hyperglycemic intrauterine environment, which may in turn impair ANS function in the offspring. Altered ANS activity can be assessed through measures such as HRV.

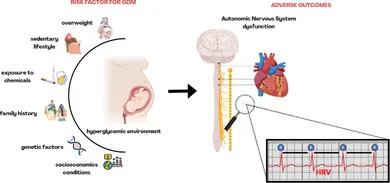

## 1 Introduction

Gestational diabetes mellitus (GDM) is defined as glucose intolerance first detected during pregnancy and is one of the most common conditions that can lead to pregnancy complications (American Diabetes Association 2003). GDM results from insufficient insulin production by pancreatic β‐cells and increased insulin resistance in tissues. Normally, insulin resistance increases in pregnancy to provide nutrients to the fetus, and β‐cells compensate by producing more insulin. However, when they fail to adapt, persistent high blood sugar levels lead to GDM (Lim et al. 2023).

Since 1995, Barker has hypothesized the influence of the intrauterine environment on fetal programming (Barker 1995). Today, the influence on the long‐term programming of life processes is increasingly recognized. It has been shown that adverse maternal conditions, such as gestational diabetes (GDM) and early postnatal life, can influence offspring development, leading to long‐term health problems in the newborn (Schlatterer and du Plessis 2021). Fetal programming, influenced by maternal hyperglycemia, stress, and inflammation, can lead to conditions like macrosomia and insulin resistance and predispose individuals to obesity, diabetes, and cardiovascular diseases later in life. Key factors include placental adaptations, altered nutrient transport, glucocorticoid regulation, and oxidative stress. Managing maternal health, including blood glucose control, is crucial to prevent adverse outcomes and reduce the risk of long‐term diseases in the offspring (Mitanchez 2010).

GDM can alter fetal and neonatal physiology through a range of pathophysiological mechanisms that might impact both heart rate variability (HRV) (Sharma et al. 2022). HRV, influenced by autonomic modulation, represents a non‐invasive tool to indirectly assess autonomic function at the cardiac level in newborns, although it does not provide a direct quantification of sympathetic or parasympathetic neural discharge (Statello et al. 2021). Consequently, HRV may be considered an indicator of early alterations in ANS development (Seifert et al. 2014; Chiera et al. 2020). In cases of pathology, evidence shows that HRV patterns are frequently altered and are often characterized by reduced HRV (Ali and Chen 2023; Agorastos et al. 2023). These variations in heart rate (HR) reflect potential early dysregulation in the balance between sympathetic and parasympathetic activity.

A reduced HRV has often been interpreted as reflecting altered autonomic balance, sometimes attributed to sympathetic predominance; however, the physiological interpretation of specific HRV components remains complex and context‐dependent (Olivieri et al. 2024). Moreover, the decrease in neonatal HRV is associated with conditions such as intrauterine growth restriction (IUGR), prematurity, congenital heart disease, and sudden infant death syndrome (SIDS) (Polson et al. 2006).

## 2 Epidemiology and Risk Factors

GDM prevalence is rising globally (Sweeting et al. 2022). In 2017, the International Diabetes Federation (IDF) estimated that 14% of global births were complicated by GDM, representing 20 million births annually (Wang et al. 2022). By 2021, this number had risen to 21.1 million, or 16.7% of births. Over 90% of cases occur in low‐ and middle‐income countries due to limited access to maternal care. In Italy, in 2021, GDM affected 14.3% of pregnancies, with an estimated 40,000 cases annually out of 400,000 pregnancies (Plows et al. 2018).

Key risk factors for GDM include being overweight, having a history of GDM or infants with macrosomia, a family history of diabetes, and coming from regions with high diabetes prevalence (Zhang et al. 2021). About 10% of women with GDM show clinical, immunological, and genetic characteristics of autoimmune diabetes, such as type 1 or Latent Autoimmune Diabetes in Adults (LADA), with nearly half developing autoimmune diabetes within a few years of pregnancy. While some factors are non‐modifiable, others, like weight, can be managed through lifestyle changes.

Screening is typically done between 24 and 28 weeks of pregnancy using either a one‐step (75 g glucose tolerance test) or two‐step approach (initial 50 g glucose test followed by a 100 g test if needed) (Hartling et al. 2012).

Guidelines recommend regular glucose monitoring, proper diet, regular physical activity, and insulin therapy, if necessary, to maintain safe glucose levels (American Diabetes Association 2015). Dietary modifications, tailored to individual cultural, economic, and pre‐pregnancy BMI factors, are essential. The American Diabetes Association (ADA) recommends a balanced diet with portion control, healthy fats, complex carbohydrates, and 20% protein (American Diabetes Association 2018). Physical activity is also recommended, with 30 min of moderate‐intensity exercise, 5 days per week.

Oral hypoglycemics, such as metformin and glyburide, have been explored as alternatives to insulin due to easier administration, better patient compliance, and lower costs. Metformin, classified as a class B drug by the FDA, works by suppressing hepatic gluconeogenesis and improving peripheral glucose uptake (Ryu et al. 2014). For instance, Ainuddin et al. have evaluated maternal and neonatal outcomes with metformin therapy, showing its effectiveness in improving glycemic control, limiting gestational weight gain, and reducing the incidence of macrosomia and excessive birth weight, while not increasing the risk of small‐for‐gestational‐age infants (Ainuddin et al. 2015).

## 3 Fetal and Neonatal Consequences of GDM

Uncontrolled GDM increases the risk of complications during pregnancy, such as fetal malformations or intrauterine death (Ornoy et al. 2021). As maternal insulin does not cross the placenta, the fetus produces its own insulin, leading to fetal hyperinsulinemia due to elevated maternal glucose levels (Sweet et al. 2013; Arshad et al. 2014). This can result in hypoxia, increased erythropoietin production, and stillbirth.

Evidence shows maternal hyperglycemia increases perinatal morbidity and mortality, with a higher risk in late pregnancy (Bianco and Josefson 2019). Fetuses exposed to maternal diabetes are at risk for congenital anomalies such as: central nervous system (CNS) malformations (caudal dysplasia, neural tube defects, macro/microcephaly), cardiac defects (transposition of the great vessels, ventricular septal defects), gastrointestinal malformations (duodenal atresia, pyloric stenosis), renal malformations (renal agenesis, hydronephrosis) (Mills 2010; Al‐Shwyiat and Radwan 2023).

The HAPO study (2008) linked maternal hyperglycemia to short‐term neonatal complications like prematurity, respiratory distress, intrauterine growth restriction, macrosomia, and metabolic disturbances like hypoglycemia and hyperbilirubinemia (HAPO Study Cooperative Research Group et al. 2008). Macrosomia increases the risk of delivery complications, while neonatal congenital heart defects are seen in 3%–6% of cases, often due to hypertrophic cardiomyopathy, which usually resolves after birth (Mitanchez et al. 2015).

**FIGURE 1:**
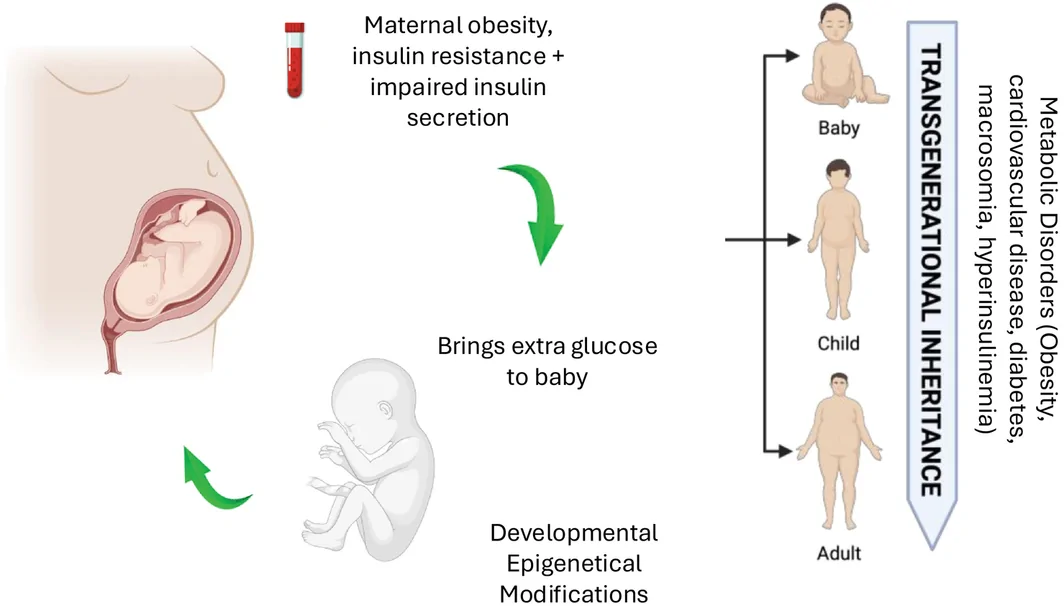
Figure 2 shows the metabolic and epigenetic impact of GDM on perinatal and offspring health.

Long‐term consequences of GDM include an increased risk of type 2 diabetes, obesity, and cardiovascular diseases in offspring (Chen et al. 2010; Sheiner 2020). Exposure to a hyperglycemic environment can affect fetal development, potentially impacting the autonomic nervous system (Russell et al. 2016) (Figure 1).

## 4 Pathophysiological Mechanisms Linking GDM With ANS

### 4.1 Physiological Development of the Autonomic Nervous System

The ANS undergoes progressive maturation throughout fetal life, reaching a functional organization near term that allows coordinated cardiovascular, respiratory, and metabolic adaptation at birth.

During fetal life, the ANS typically matures by 37 weeks of gestation and adapts to new environmental challenges at birth (Gagnon et al. 1987). ANS is responsible for regulating HRV; in turn, HRV monitoring serves as a good indicator of general neonatal well‐being, capable of detecting complications related to pregnancy and birth (Seifert et al. 2014; Chiera et al. 2020; Oliveira et al. 2019). Under physiological conditions, this maturation reflects a gradual increase in autonomic complexity, characterized by dynamic interplay between sympathetic activation, supporting energy mobilization and adaptation to stress, and parasympathetic modulation, promoting regulation, recovery, and homeostasis (Oliveira et al. 2019).

### 4.2 Metabolic and Inflammatory Stressors in GDM

In pregnancies complicated by GDM, the intrauterine environment is characterized by persistent metabolic stressors, including maternal hyperglycemia, fetal hyperinsulinemia, oxidative stress, and low‐grade inflammation (Plows et al. 2018).

These factors represent chronic biological stressors during a critical window of ANS maturation, potentially interfering with the normal trajectory of autonomic development.

**FIGURE 2:**
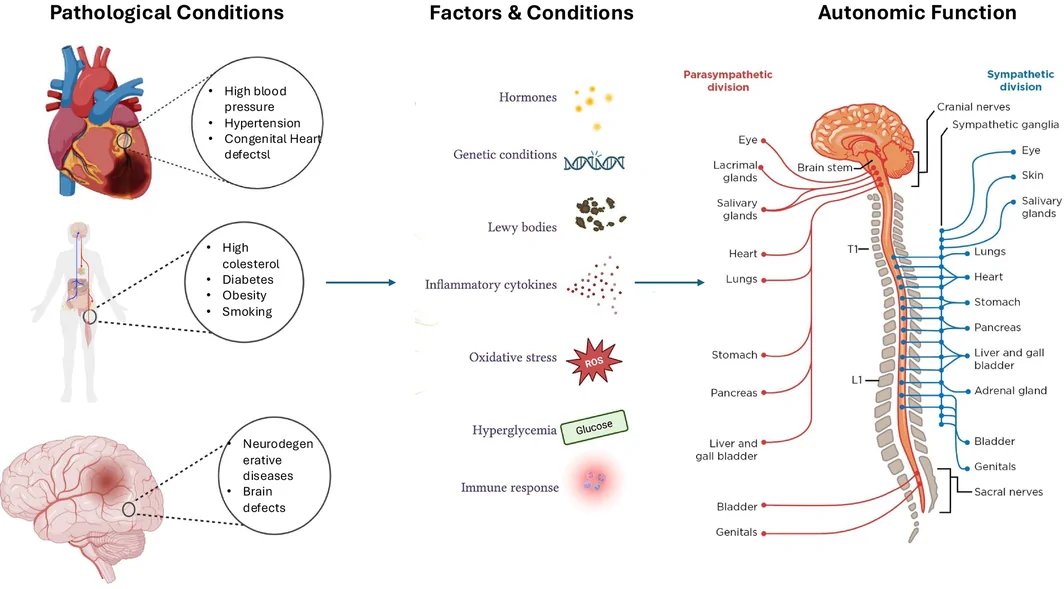
Depicts how GDM, through hyperglycemia, inflammatory cytokines, oxidative stress, and immune activation, has an impact on ANS regulation and activity. These mechanisms contribute to modified autonomic balance in the fetus and newborn, potentially reflected by changes in heart rate variability.

The hyperglycemic environment associated with GDM can lead to chronic inflammation and oxidative stress, both of which may impact fetal ANS development (Saucedo et al. 2023). The ANS is sensitive to metabolic alterations, and prolonged poor glycemic control is known to be associated with autonomic neuropathy in adult patients with diabetes (Spallone 2019). Figure 2 shows how these mechanisms contribute to modified autonomic balance in the fetus and newborn, potentially reflected by changes in heart rate variability.

### 4.3 Mechanistic Pathways Linking Hyperglycemia to Altered ANS Development

Mechanistically, prolonged exposure to hyperglycemia may alter autonomic maturation through multiple pathways. Elevated glucose levels can enhance oxidative stress and inflammatory signaling, which may affect brainstem nuclei involved in autonomic regulation. In addition, fetal hyperinsulinemia may influence energy metabolism and neuroendocrine signaling, potentially modifying sympathetic‐parasympathetic balance during critical developmental periods (Vogt et al. 2014).

Furthermore, altered placental function and vascular reactivity may contribute to subtle hypoxic or metabolic fluctuations, further acting as modulators of autonomic circuit development.

Studies have demonstrated altered HRV patterns in infants born to mothers with GDM, potentially signifying an early ANS imbalance (Sharifi‐Heris et al. 2023). In 2016, Fehlert et al. assessed ANS activation during oral glucose loading using maternal monitoring and magnetocardiography (fMCG) and demonstrated that fetuses of mothers with GDM showed reduced overall HRV and an altered sympathovagal balance during oral glucose loading, characterized by exaggerated sympathetic responsiveness compared to fetuses of normoglycemic mothers (Fehlert et al. 2017). Furthermore, a longitudinal cohort study conducted by Zöllkau et al. analyzed the relationship between maternal metabolic control and autonomic regulation of fetal heart rate, demonstrating that both sympathetic and vagal activity are influenced by GDM (Zöllkau et al. 2021). In this study, monthly fMCG was performed at 27, 31, 35, and 39 weeks of gestation with beat‐to‐beat heart rate recording for heart rate variability analysis. The results demonstrated that in the diabetic population, sympathetic activation was associated with higher maternal blood glucose levels. On the other hand, after 32 weeks of gestation, higher fetal insulin levels also appear to be associated with increased vagal modulation during the active sleep state.

Another recent study revealed clear associations between maternal body mass index (BMI), glycosylated hemoglobin (HbA1c), and fetal HR, with a negative correlation between HRV and BMI/HbA1c and a positive correlation between fetal heart rate and BMI/HbA1c (Chivers et al. 2025). These results indicate that poorer maternal glycemic control is associated with a reduced ability of the ANS to modulate heart rate, as well as a tendency toward higher heart rates caused by sympathetic activation.

Most studies investigating ANS function during pregnancy have primarily focused on cardiovascular parameters. However, the fetal period has also garnered attention in recent years. In this context, Hamoud et al. analyzed HRV in fetuses, comparing 26 fetuses of mothers with type 1 diabetes to 26 fetuses of non‐diabetic mothers. The study revealed significant alterations in HRV patterns, suggestive of an increase in parasympathetic activity in the diabetic group (Hamoud et al. 2019).

A review by Van Laar et al. further contributed to our understanding of autonomic modulation during pregnancy. The authors found a progressive decrease in the LF/HF ratio as gestation advances, hypothesizing that the increase in high‐frequency (HF) power—representative of parasympathetic activity—outpaces that of low‐frequency (LF) power. This shift, especially evident beyond the 30th week of gestation and persisting until delivery, indicates a gradual decline in sympathetic dominance in favor of parasympathetic control (Van Laar et al. 2008) In neonatal period HRV studies have often considered results derived from comparisons between preterm and term newborns, which clearly suggest that preterm newborns exhibit ANS underdevelopment (Javorka et al. 2017).

The impact of maternal metabolic conditions on neonatal autonomic regulation has also been the subject of investigation. Newborns exposed to gestational diabetes mellitus (GDM) may exhibit altered HRV, reflecting disrupted autonomic balance (Russell et al. 2016; Gardim et al. 2014). In particular, Russell et al. found that infants born to diabetic mothers presented with a higher LF/HF ratio in postnatal ECG analyses, suggesting sympathetic overactivity compared to those born to normoglycemic mothers.

### 4.4 From Fetal Programming to Postnatal Autonomic Adaptation

The transition from intrauterine to extrauterine life represents a major physiological stress test for the newborn. Under normal conditions, this transition is characterized by a coordinated activation of both sympathetic and parasympathetic systems, allowing cardiovascular stabilization, thermoregulation, and metabolic adaptation.

In infants exposed to GDM, however, this adaptive response may be altered. Chronic intrauterine metabolic exposure could lead either to exaggerated sympathetic priming or, conversely, to reduced sympathetic reserve due to sustained prenatal activation. Such patterns may manifest as altered autonomic modulation in the immediate postnatal period (Weiss et al. 2024).

In a more recent study, Ravenda et al. (2025) monitored heart rate in 64 full‐term newborns during the first hours of extrauterine life (Ravenda et al. 2025). The study demonstrated that infants born to mothers with GDM exhibited significantly lower heart rates and higher vagally‐mediated HRV indices than controls. Interestingly, the control group showed greater sympathetic activation, likely reflecting a robust physiological response to the stress of birth. Conversely, the blunted sympathetic response in the GDM group may suggest a reduced adaptive capacity to perinatal stress.

This phenomenon can be interpreted as a form of intrauterine preconditioning. Chronic exposure to hyperglycemia and other metabolic stressors in utero may lead to early sympathetic system activation, exhausting its capacity and limiting further responsiveness after birth. As a result, these neonates may rely more heavily on parasympathetic regulation in the immediate postnatal period.

The transition from intrauterine to extrauterine life requires substantial physiological adjustments, including cardiovascular, endocrine, respiratory, and thermoregulatory changes. During this critical period, both branches of the ANS, sympathetic and parasympathetic, are activated, along with brainstem and supratentorial structures such as the hypothalamus and forebrain, to support neonatal adaptation (Mulkey and du Plessis 2019).

In general, higher baseline HRV values, particularly those associated with vagal tone, are correlated with better health outcomes and lower stress levels in both adults and neonates (Thayer et al. 2012). In newborns of diabetic mothers, however, the apparent parasympathetic predominance observed after birth may not necessarily reflect a state of well‐being. Rather, it could signal an impaired sympathetic reserve due to prenatal overactivation, limiting the neonate's ability to mount an adequate physiological response to postnatal challenges.

Neuroendocrine regulation represents an additional layer of interaction between metabolic programming and autonomic adaptation. The hypothalamic–pituitary–adrenal (HPA) axis and the autonomic nervous system operate in close functional integration during the perinatal transition (Sheng et al. 2021).

In pregnancies complicated by GDM, chronic intrauterine metabolic exposure can influence fetal HPA axis programming and postnatal stress reactivity (Sheng et al. 2021). Altered cortisol dynamics may therefore interact with autonomic regulation, contributing to the modified HRV patterns observed in exposed neonates.

Within a developmental programming framework, these findings suggest that neuroendocrine and autonomic systems should be considered jointly when interpreting early postnatal HRV alterations.

## 5 Critical Consideration on HRV Methodology

Although heart rate variability is widely employed as a surrogate marker of autonomic nervous system activity, its interpretation requires methodological caution. HRV reflects fluctuations in the sinoatrial node firing pattern resulting from complex interactions between sympathetic and parasympathetic influences, intrinsic cardiac properties, baroreflex mechanisms, respiratory patterns, and central regulatory inputs (Shaffer et al. 2014). Therefore, HRV indices do not provide a direct measurement of neural sympathetic or vagal traffic.

Time‐domain indices such as SDNN represent overall variability and are strongly influenced by parasympathetic modulation (Shaffer et al. 2014). Experimental vagal blockade with atropine has demonstrated a marked reduction of SDNN, highlighting the predominant role of vagal activity in short‐term recordings (Liu et al. 2020). Conversely, frequency‐domain measures, particularly low‐frequency power, have historically been interpreted as reflecting sympathetic activity; however, evidence from both pharmacological blockade studies and pathological conditions (e.g., chronic heart failure) indicates that low‐frequency power cannot be considered a pure index of sympathetic tone (Liu et al. 2020; Hopf et al. 1995). In Table 1 are summarized these HRV indices and their characteristics.

**TABLE 1:** Commonly used HRV measures and their physiological interpretation.

HRV index	domain	Conventional interpretation	Methodological considerations
SDNN	Time‐domain	Overall variability	Strong vagal contribution in short recordings
RMSSD	Time‐domain	Parasympathetic (vagal) modulation	Highly sensitive to respiration
HF power	Frequency‐domain	Parasympathetic activity	Influenced by breathing frequency
LF power	Frequency‐domain	Mixed sympathetic‐parasympathetic influence	Not a pure sympathetic marker
LF/HF ratio	Frequency‐domain	“Sympathovagal balance” (controversial)	Oversimplified interpretation
Nonlinear indices (e.g., entropy)	Nonlinear	System complexity and adaptability	Sensitive to recording quality

Importantly, sympathetic and parasympathetic systems do not operate in a strictly reciprocal manner. Their interaction is dynamic, nonlinear, and influenced by developmental stage, metabolic state, and environmental stressors, particularly relevant in fetal and neonatal physiology (Shaffer et al. 2014; Hopf et al. 1995).

Moreover, neonatal HRV interpretation is further complicated by the developmental immaturity of autonomic circuits, differences in sleep state organization, respiratory instability, and variability in recording length and signal preprocessing methods (Latremouille et al. 2021). Differences in ECG sampling rate, artifact correction, epoch duration, and analytical approach (time‐domain vs. frequency‐domain vs. nonlinear metrics) may significantly influence results across studies.

For these reasons, HRV should be interpreted as an indirect indicator of autonomic modulation rather than a direct quantification of sympathetic or parasympathetic activity. Careful consideration of methodological parameters is essential when translating HRV findings into clinical practice.

## 6 Integrative Perspective

In summary, normal ANS development is characterized by progressive maturation and increasing regulatory flexibility, enabling effective adaptation to environmental and physiological challenges. In the context of GDM, the fetus is exposed to chronic metabolic and inflammatory stressors during a critical window of autonomic organization. These stressors may interfere with brainstem regulatory circuits, neuroendocrine balance, and cardiovascular control mechanisms, potentially leading to altered autonomic modulation at birth (Weiss et al. 2024).

Rather than representing a simple shift toward sympathetic or parasympathetic dominance, the observed HRV alterations in GDM‐exposed newborns likely reflect modified autonomic adaptability. Understanding this distinction is essential for interpreting neonatal HRV findings and for framing GDM as a condition capable of influencing early‐life stress physiology and long‐term cardiometabolic risk.

## 7 Autonomic Nervous System and Gender Differences

Gender differences in ANS indexes are well documented in pediatric and adult populations (Koenig and Thayer 2016; Koenig et al. 2017; Faulkner et al. 2003; Moodithaya and Avadhany 2012). These studies have revealed significant differences in heart rate and HRV. Males typically show lower HRV indexes compared to females, likely due to increased sympathetic activity leading to reduced vagal modulation of the heart (Koenig and Thayer 2016; Koenig et al. 2017).

This may contribute to greater cardiovascular reactivity and reduced adaptability to environmental stressors (Faulkner et al. 2003; Moodithaya and Avadhany 2012). Females, on the other hand, show higher vagal modulation, which is associated with improved stress resilience and cardiovascular regulation (Zhang et al. 2023). In newborns from mothers with GDM, these gender differences may be further amplified or modified due to prenatal exposure to diabetes. Analyzing HR in full‐term infants, while there were no differences in HR and HRV between males and females in newborns of healthy mothers, there were significant differences in the GDM group, suggesting a clear influence of GDM on cardiac autonomic function (Ravenda et al. 2025). Indeed, females with GDM showed significantly lower HR values than females within the control group, while no difference was found between males within the GDM group and the control group.

In this context, understanding how gender differences can influence neonatal adaptation and response to environmental stimuli from the intrauterine to extrauterine environment is critical. Gender‐informed therapeutic strategies targeting autonomic regulation could improve neonatal outcomes. For example, vagal stimulation techniques might be more beneficial for males, while stress‐reduction approaches could optimize outcomes for both sexes (Task Force of the European Society of Cardiology and the North American Society of Pacing and Electrophysiology 1996). Developing a more targeted approach to risk stratification and personalized treatment is essential for optimizing therapeutic regimens, minimizing inappropriate treatments, and improving cost‐effectiveness.

## 8 Neuroendocrine Regulation and Clinical Implications

Stress is a complex process regulated by two main neural systems: the HPA with cortisol as a classical stress biomarker and the autonomic nervous system with HRV as a recently suggested stress marker. Several studies have explored the relationship between cortisol responses and HRV, notably the cortisol awakening response, which has been investigated in both children and adults (Nader and Weems 2011; Michels et al. 2013).

In pediatric populations, elevated cortisol levels have been consistently associated with HRV patterns indicative of reduced parasympathetic modulation, suggesting an inverse relationship between these two systems under stress conditions (Michels et al. 2013).

In the neonatal period, stress regulation undergoes profound changes. Our previous findings demonstrated a significant decline in cortisol levels between 8 and 15 h after birth, even in neonates born to mothers with GDM (Ravenda et al. 2025). This early postnatal decrease in cortisol may reflect the activation of oxytocinergic neurons shortly after birth, a mechanism that has been shown to lower HR and enhance vagal tone (Rogers and Hermann 1986; Higa et al. 2002). These findings align with the broader evidence supporting the role of early maternal contact, including skin‐to‐skin interaction and prolonged Kangaroo care, in promoting physiological stability and resilience in newborns (Durmaz et al. 2023; Cristóbal Cañadas et al. 2022). Such positive early exposures appear to buffer the effects of stress and support optimal neurodevelopmental trajectories.

The observed reduction in cortisol levels within the first 15 h of life can be interpreted as a protective adaptation. Persistently elevated cortisol concentrations in neonates have been linked to long‐term negative outcomes, including heightened emotional reactivity and less adaptive stress responses later in life (McLean et al. 2023). Maternal contact during the early postnatal period, through practices such as rooming‐in, may function as an external regulatory input, modulating cortisol secretion via both the HPA axis and the ANS.

Corticosteroids play a critical role in fetal development, particularly in preparing newborns for life outside the womb. Before birth, there is an increase in sympathetic nervous system activity and a neuroendocrine response, characterized by elevated levels of catecholamines, cortisol, thyroid hormones, and components of the renin‐angiotensin system (Mulkey and du Plessis 2019; Morton and Brodsky 2016).

The transition from intrauterine to extrauterine life involves complex hormonal and neurophysiological processes. Corticosteroids play a crucial role in fetal maturation, preparing multiple organ systems for independent function at birth (Perrone et al. 2023). This preparatory phase is characterized by an upregulation of sympathetic activity and a robust neuroendocrine response, marked by elevated levels of catecholamines, cortisol, thyroid hormones, and elements of the renin‐angiotensin system (Mulkey and du Plessis 2019; Morton and Brodsky 2016). While these mechanisms are vital for survival, dysregulation—such as persistently high cortisol—has been correlated with increased temperamental negativity in infants and reduced vagal tone, the latter being a known risk factor for cardiovascular and metabolic diseases later in life. Studies in infants at 12 weeks show that higher baseline cardiac vagal tone is associated with fewer negative behaviors, while those with reduced vagal tone exhibit more negative temperament traits (Huffman et al. 1998).

Importantly, cortisol not only mediates the stress response but is also associated with increased HR. In contrast, oxytocin, often released during maternal–infant interactions, has been shown to produce opposing effects, reducing HR and promoting parasympathetic activity. This balance between cortisol and oxytocin reflects the dynamic interplay between stress and social bonding systems, both of which are critically engaged during the perinatal period (De Bernardo et al. 2018).

The significant reduction in cortisol levels from 8 to 15 h of life even in newborns from GDM mothers, further supporting the notion that early maternal–infant contact, particularly skin‐to‐skin and Kangaroo care, plays a protective role in the development of stress regulatory systems (De Bernardo et al. 2018). These practices appear to enhance resilience, defined as the capacity to maintain physiological and emotional stability in the face of stress. Conversely, early maternal–infant separation has been associated with increased stress reactivity, impaired bonding, and a heightened risk for both emotional and physical health issues (De Bernardo et al. 2018).

The neuroscience of birth and breastfeeding underscores the importance of early affective bonding and mutual regulation between mother and infant. This co‐regulation fosters secure attachment, facilitates the initiation and maintenance of breastfeeding, and supports the maturation of neurobiological systems involved in stress resilience. On the other hand, disruption of this early dyadic interaction can promote toxic stress, attachment disturbances, and greater vulnerability to illness.

Hormonal systems, particularly oxytocin and cortisol, play central roles during the perinatal period, orchestrating the newborn's adaptation to extrauterine life. While oxytocin is associated with social bonding and calming effects, cortisol enables the newborn to cope with the stress of birth. The balance and interaction of these hormones may serve as early predictors of later health outcomes.

Analyzing both HRV and salivary cortisol as markers of autonomic and adrenal function in newborns of mothers with GDM during the immediate postnatal period offers novel insights into how early maternal care and hormonal regulation interact to shape the development of stress physiology and may guide future interventions aimed at promoting resilience from the very beginning of life.

## 9 Conclusion

The early identification of HRV in newborns from mothers with GDM can be critical in preventing adverse long‐term health outcomes. Neonatal HRV may serve as non‐invasive biomarkers for the early detection of metabolic or cardiovascular risk, which are prevalent concerns in offspring of mothers with GDM. By identifying infants at risk, targeted intervention strategies can be implemented, such as dietary modifications and stress management techniques, potentially improving cardiovascular and metabolic outcomes in the long term.

The integration of HRV into neonatal follow‐up could offer a valuable approach to stratifying risk and tailoring care to the individual needs of neonates exposed to GDM. Moreover, continuous follow‐up on this parameter throughout early childhood could provide insights into the effectiveness of interventions and support a preventative approach to long‐term health maintenance.

## Author Contributions

V.B.: conceptualization, methodology, visualization, writing‐original draft, writing‐review and editing; L.C.: supervision, writing‐review and editing; S.R.: visualization; C.P.: supervision, visualization; S.M.: supervision, visualization; V.D.: visualization; S.P.: conceptualization, supervision, visualization, writing‐review and editing.

## Funding

Work supported by #NEXTGENERATIONEU (NGEU) and funded by the Ministry of University and Research (MUR), National Recovery and Resilience Plan (NRRP), Project MNESYS (PE0000006) – A multiscale integrated approach to the study of the nervous system in health and disease (DN. 155311.10.2022).

## Conflicts of Interest

The authors declare no conflicts of interest.

## Data Availability

The authors have nothing to report.
